# A93 USEFULNESS OF A NOVEL SMARTPHONE APP IN GASTROINTESTINAL ENDOSCOPY TO TRACK PROCEDURE NUMBERS AND THERAPEUTIC INTERVENTIONS

**DOI:** 10.1093/jcag/gwab049.092

**Published:** 2022-02-21

**Authors:** D Y Yang, T Krahn, C Wang, J Decanini-Trevino, S Wasilenko, K Kroeker, A Davila-Cervantes, D C Baugmart, A J Montano-Loza, B Halloran, S Zepeda-Gomez

**Affiliations:** 1 Medicine, University of Alberta, Edmonton, AB, Canada; 2 Gastroenterology, University of Alberta, Edmonton, AB, Canada; 3 University of Alberta, Edmonton, AB, Canada; 4 Division of Gastroenterology & Liver Unit, University of Alberta, Edmonton, AB, Canada

## Abstract

**Background:**

Endoscopy teaching is an integral part of gastroenterology (GI) training. Though the number of completed endoscopic procedures does not equate competency, procedure tracking is useful for monitoring an individual’s learning progress. Currently, procedure tracking is typically done on an informal basis using paper or electronic spreadsheets. These methods are non-standardized and may not be shareable between trainees and their programs.

Endostation is a smartphone app created by the University of Alberta Therapeutic Endoscopy Program to facilitate the tracking of endoscopic procedures. The app allows trainees to record the number of endoscopies and details such as cecal intubation (CI), ERCP cannulation, and therapeutic interventions. Data can be accessed by users via the app and website (www.endostation.ca), allowing for close monitoring of trainees’ learning progress.

**Aims:**

Our primary objective was to evaluate the usefulness of the app for tracking the number of endoscopic procedures and therapeutic interventions. Our secondary objective was to evaluate the acquisition of endoscopy skills based on quality endoscopic parameters such as CI rate and ERCP cannulation rate.

**Methods:**

One therapeutic endoscopy fellow and two GI residents were recruited for the study. Participants were asked to document their procedures over the study period (9-month for therapeutic endoscopy fellow, 12-month for GI residents). Total number of procedures was summed for each trainee. Acquisition of endoscopy skills was tracked by comparing success rates of CI and ERCP cannulation at different points within the study period.

**Results:**

The therapeutic endoscopy fellow recorded 415 cannulation attempts, 209 sphincterotomies, 282 stone extractions, 71 plastic stent placements, and 37 metal stent placements. There was a significant difference in the cannulation success rate when comparing the 1^st^ trimester and the 3^rd^ trimester of the study period (68% vs 85%; p= 0.0012) (Fig 1).

The two GI residents respectively recorded 335 and 170 colonoscopies plus 454 and 305 gastroscopies. Resident 1 recorded 58 polypectomies, 9 esophageal variceal banding, and 16 non-variceal hemostasis. Resident 2 recorded 17 polypectomies, 12 esophageal variceal banding, and 9 non-variceal hemostasis. The CI success rate was significantly higher for both residents when comparing the first 4 months of training vs the last 4 months [24% vs 88% for resident 1 (p=0.00001); 15% vs 42% for resident 2 (p= 0.001)] (Fig 1).

**Conclusions:**

The smartphone app (Endostation) was a useful tool for endoscopic procedure tracking. Data from the app was useful in demonstrating improvement in CI rate and ERCP cannulation rate over the study period.

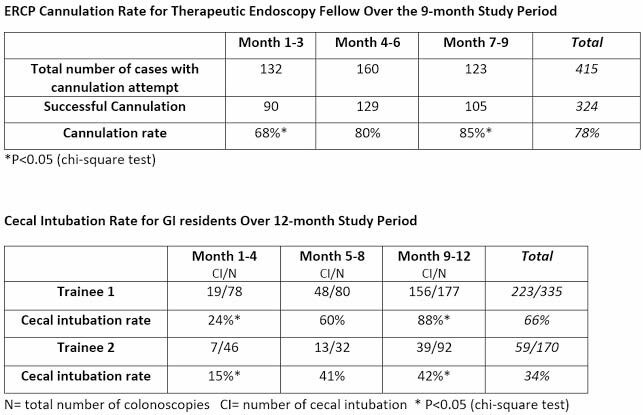

**Funding Agencies:**

None

